# Recent advances and challenges of cellular immunotherapies in lung cancer treatment

**DOI:** 10.1186/s40164-025-00679-8

**Published:** 2025-07-07

**Authors:** Chengfei Yang, Yue Liu, Ziqi Huang, Sijin Liu, Xi Zhang, Quanxing Liu, Jigang Dai

**Affiliations:** 1 Department of Thoracic Surgery, Xinqiao Hospital, Army Medical University (Third Military Medical University), Chongqing, 400037 China; 2 Medical Center of Hematology, State Key Laboratory of Trauma and Chemical Poisoning, Chongqing Key Laboratory of Hematology and Microenvironment, Xinqiao Hospital of Army Medical University, Chongqing, 400037 China

**Keywords:** Cellular immunotherapy, Lung cancer, CAR-T therapy, Antigen targets, Tumor microenvironment, Combination immunotherapy strategy

## Abstract

Lung cancer is a major malignant tumor with high morbidity and fatality rates. For many years, traditional treatments for lung cancer have struggled to achieve a favorable outlook and prognosis. It is crucial to identify and innovate novel clinical therapeutic strategies and techniques to prevent tumor progression and prolong the survival time of patients with lung cancer. Cellular immunotherapies have revolutionized the treatment of malignant tumors and have been gradually applied in clinical practice. CAR-T therapy is the best-known cellular therapy and has achieved remarkable clinical outcomes in patients with hematological malignancies, but its effect on patients with lung cancer and other solid tumors is not satisfactory, partly because of the heterogeneity and complexity of lung cancers and the sterile TMEs. To further improve the clinical effect, multiple approaches and strategies have been adopted, including discovering new tumor antigen targets, improving safety, enhancing cytotoxicity, and increasing durability. Moreover, other cell-based immunotherapies have also showed great potential for the treatment of lung cancer, including TCR-T cells, TILs, CIK cells, NK cells, macrophages, and dendritic cells, which enriched the number of treatment choices for patients with lung cancer. In summary, the present article summarizes and highlights recent advances and challenges in the use of cellular immunotherapies for the treatment of lung cancer, which might stimulate new ideas for the further development of cellular immunotherapies.

## Background

Lung cancer is a common malignant tumor with high morbidity and fatality rates in both male and female [1, 2]. According to the latest global cancer burden research from the World Health Organization (WHO) in 2024, there were approximately 2.48 million new cases of lung cancer, representing approximately one-eighth of all cancer cases [3, 4]. The number of deaths caused by lung cancer reached up to 1.82 million, accounting for 18.7% of all cancer deaths worldwide, making lung cancer the leading cause of cancer deaths [3]. The two major types of lung cancer are small cell carcinoma (SCLC) and non-small cell carcinoma (NSCLC). NSCLC accounts for approximately 85% of all diagnosed lung cancers [2, 5, 6]. For many years, the main treatment strategy for lung cancer is surgical resection combined with several adjuvant therapies, including chemotherapy, radiotherapy and targeted therapy [7]. However, the 5-year survival rate is still lower than 20% [8, 9]. More than 75% of patients with lung cancer are diagnosed in advanced stages and miss the opportunity for radical surgery [10, 11]. Therefore, it is critical to identify and innovate clinical therapeutic strategies to prevent tumor progression and prolong the survival time of patients with lung cancer.

Immunotherapy has been gradually applied to treat patients with cancer and has shown promising clinical benefits in recent decades [12–16]. This therapy can reactivate an individual’s own immune system to attack cancer cells and has been shown to affect all tumors. For NSCLC, more than 10 immune checkpoint inhibitors targeting programmed death-1 (PD-1) and programmed death-ligand 1 (PD-L1) have been approved for clinical therapy [17–19]. The pharmacologic inhibition of PD-1/PD-L1 aims to disrupt the interaction between tumor cells and T cells, thereby eliminating this immunosuppressive mechanism and restoring cytotoxic T cell activity [20, 21]. In clinical trials of pembrolizumab as a first-line treatment, the objective response rate (ORR) was greater than 45% of patients with advanced-stage NSCLC [22, 23]. Moreover, a CTLA-4 inhibitor has also been approved for the treatment of NSCLC, and the combination therapy with PD-1 inhibitors has resulted in a relatively high response rate and prolonged overall survival time [17, 24–26].

In recent years, adoptive cellular immunotherapies have displayed enhanced anti-tumor efficacy, especially chimeric antigen receptor T (CAR-T) cell therapy [27, 28]. CAR-T therapy was initially developed and used to treat hematological malignancies, and it has achieved great curative effects [29–32]. More than 10 CAR-T cell products have been commercially approved for the treatment of leukemia, lymphoma, and myeloma. The complete remission (CR) rate in patients with relapsed/refractory acute B-lymphocytic leukemia treated with CD19 CAR-T is 30–70% and even exceeds 90% in some trials [33, 34]. The promising clinical efficacy of CAR-T therapy for the treatment of hematological malignancies have increased its application for the treatment of solid tumors with many clinical trials currently being conducted worldwide. However, its clinical efficacy in treating solid tumors is far less than that in treating hematological malignancies, which is partly because of the limited tumor antigen targets, impaired T cell function, and sterile tumor microenvironments (TMEs) [35–38]. In this review, we summarize the recent advances and challenges in the use of cellular immunotherapies, including CAR-T cell therapy and other immune cell-based therapies for the treatment of lung cancer. Furthermore, we aim to address the major difficulties and provide ideas for future prospects of cellular immunotherapies for the treatment of lung cancer.

## The development and evolution of CAR-T therapy

**Fig. 1:**
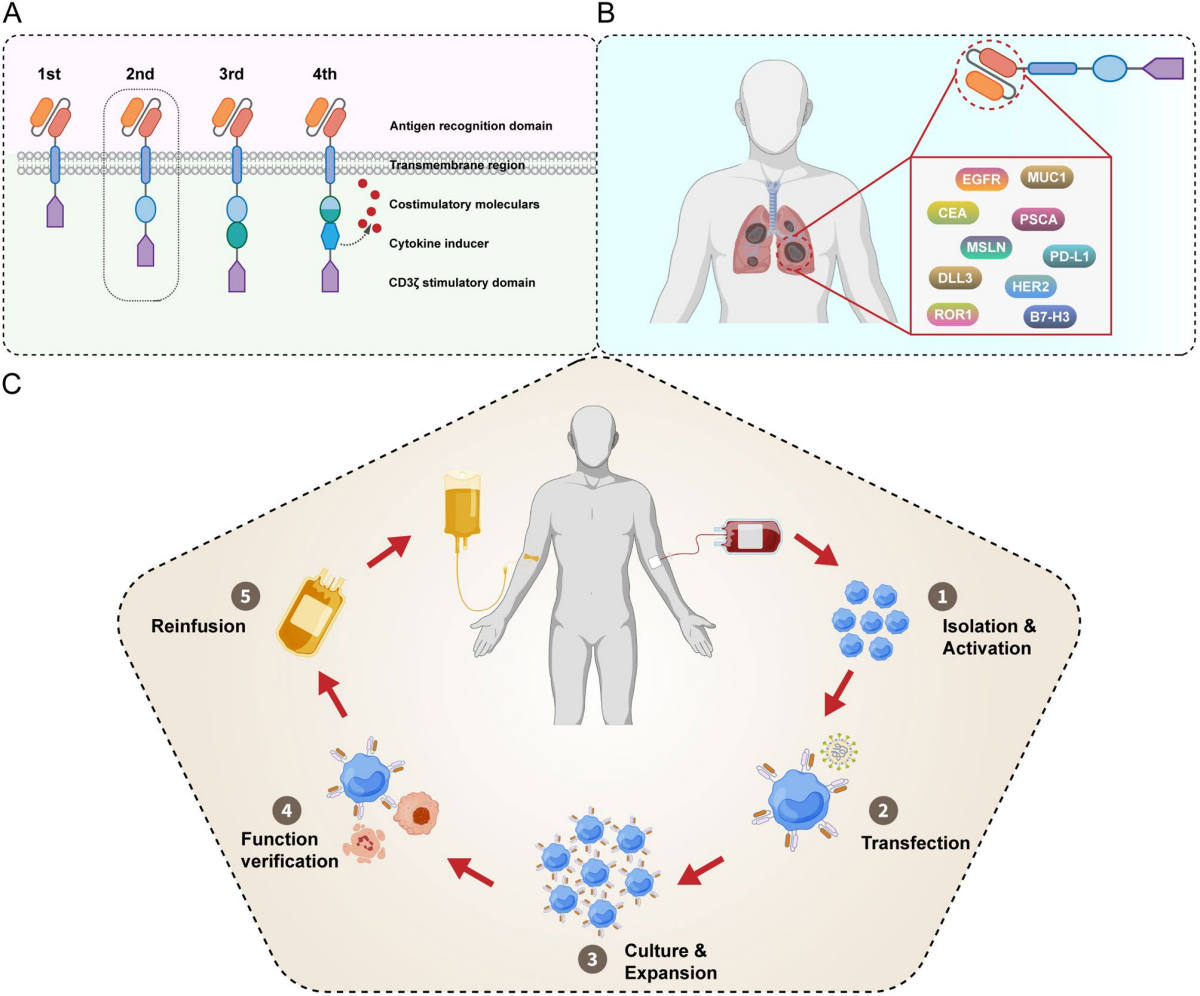
**A** The schema of construction of 1^st^, 2^nd^, 3^rd^, and 4^th^ generation CARs. **B** The potential targets for CAR-T cells for the treatment of lung cancer. **C** An overview of the CAR-T cell therapy process

The CAR concept was initially proposed in 1989, when G Gross et al. designed a chimeric receptor protein containing an intracellular signaling domain from T cell receptor (TCR) and an antibody variable domain, and it was added to T cell to activate cytotoxicity [29]. The artificial protein was the prototype of CAR and subsequently the receptor structure undergone continuous optimization and evolution [39]. Costimulatory domains (e.g., CD28, 4-1BB, and OX40) were inserted into the intracellular region to enhance the anti-tumor cytotoxicity, the proliferation ability, and the durability of CAR-T cells, regarded as the second-generation CAR structure [40–42]. Notably, CAR-T cells based on the second-generation structure are the most widely used one in preclinical experiments and clinical trials. The second-generation CAR structure composed of an extracellular antigen recognition domain, an extracellular spacer, a transmembrane domain, an intracellular signaling domain, and a costimulatory domain (Fig. 1A). Then, several proinflammatory cytokines (e.g., IL-2, IL-12, IL-15, and IL-18) and chemokines have been sequentially fused onto the CAR structure [43–46]. The participation of these cytokine and chemokine signals promotes the infiltration of CAR-T cells into tumor tissue, especially the tissues of solid tumors. Moreover, recent studies have introduced artificial heterozygous receptors, including an extracellular inhibitory motif and an intracellular stimulatory motif, such as TGF-β/IL-7, PD-1/CD28, and CTLA-4/CD28, into CAR-T cells [47–50]. These chimeras are capable of delivering activated signals rather than inhibitory signals and significantly enhanced CAR-T reactivity. Undoubtedly, these continuous remoulding and improving of the CAR structure increase the potential efficacy of CAR-T cell therapy.

The conventional preparation of CAR-T cells is divided into five steps: (1) The isolation and activation of T cells from patients; (2) CAR structure preparation and genetic manipulation; (3) CAR-T cells culture and expansion; (4) CAR-T cell phenotype detection and function verification; (5) CAR-T cells reinfusion into patients (Fig. 1C). For traditional CAR-T therapy, the T cell material is usually obtained from the patients themselves, which results in an inevitable time cost approximately three to four weeks for preparation. In addition, quality and function tend to be impaired because of the long-term infiltration and suppression in the TME [51]. To overcome these problems, allogeneic CAR-T cells have emerged. These cells originated from healthy donors and can be collected, manufactured, and detected in advance and then infused into patients without delay. Compared with patient T cells, healthy donor T cells exhibit enhanced anti-tumor ability [52, 53]. In addition, the systematic large-scale and convenient manufacturing of allogeneic CAR-T cells reduces the average cost and results in a process that is more easily carried out. Recently, a series of clinical trials of allogeneic CAR-T have shown efficient anti-tumor performance against both hematological and solid malignancies [54–58]. Allogeneic CAR-T therapy has gradually gained importance in the evolution of CAR-T therapy.

## The potential antigen targets for CAR-T for the treatment of lung cancer

Exploring and identifying specific tumor-associated antigens (TAAs) as the target is a significant step for CAR-T therapy in solid tumors. Specific or high-level expression in tumor cells together with absent or few-level expression in normal cells is the criterion for an optimal TAA. In lung cancer, several potential targets for CAR-T have been identified, including epidermal growth factor receptor (EGFR), carcinoembryonic antigen (CEA), mesothelin (MSLN), human epidermal growth factor receptor 2 (HER2), receptor tyrosine kinase-like orphan receptor 1 (ROR1), Mucin 1 (MUC1), prostate stem cell antigen (PSCA), programmed death-ligand 1 (PD-L1), B7-H3, and Delta-like ligand 3 (DLL3) (Fig. 1B), listed in Table 1.

**Table 1:** The potential antigen targets in lung cancer for CAR-T therapy

Antigen targets	Function&Properties	Expression		Relationship to lung cancer		Targeted drugs
**EGFR**	A typical human epidermal growth factor receptor; Responsible for extracellular signal transduction into cells.	Mainly expressed on the cytomembranes of epithelial cells; Highly expressed in several epithelium-derived tumors, including kidney cancer, lung cancer, prostate cancer, pancreatic cancer, and breast cancer.		Approximately 60% of patients with NSCLC have EGFR mutations. EGFR mutations induce excessive activation of lung cancer cells, resulting in sustained proliferation and invasion.		Gefitinib, Afatinib, Osimertinib, etc.
**CEA**	An acidic glycoprotein with human embryonic antigen properties; Involved in cell-to-cell adhesion by binding to intercellular adhesion molecules.	Mainly expressed in the colon during fetal development; Highly expressed in certain digestive system neoplasms, including those of colorectal cancer, lung cancer, gastric carcinoma, and pancreatic cancer.		A typical tumor marker for lung cancer; The expression is negatively correlated with tumor prognosis.		CEA/CD3 bispecific antibody
**MSLN**	A glycosylated phosphatidylinositol-anchored protein; Unclear biological functions in normal tissue.	Rarely expressed in normal tissues, except for at low levels in bronchus, fallopian tube, placenta, and tonsil; Highly expressed in tumor tissues, including mesothelioma, pancreatic cancer, lung cancer, etc.		A significant diagnostic and therapeutic target for lung cancer; Its abnormal high expression promotes lung cancer cell proliferation, local invasion, and malignant metastasis.		MSLN-ADC
**HER2**	A tyrosine kinase receptor belonging to the ERBB/HER family; Involved in signal activation and transduction, including MAPK, PI3K/AKT, and STAT pathways.	Normally expressed in the fetus, while low levels in a few tissues in adulthood; Highly expressed in epithelial originated-tumors, including breast cancer, lung cancer, gastric cancer, etc.		The mutate rate is approximately 2 - 4% and the overexpression rate is approximately 15%. The mutate and abnormal expression of HER2 promotes lung cancer cell invasion and malignant metastasis		Detrastuzumab, Zongertinib
**ROR1**	A subfamily of tyrosine kinase receptors; Involved in intercellular communication and intracellular signal transduction by binding and activating Wnt family proteins.	Normally expressed in the fetus, while gradually disappeared in most healthy tissues; Highly expressed in hematologic malignancies and solid tumors.		ROR1 is highly expressed in lung cancer and is regarded as a prognostic biomarker in patients with lung cancer. ROR1 protein promotes proliferation and migration of lung cancer cells.		Zilovertamab vedotin, Cirmtuzumab
**MUC1**	A transmembrane mucin and glycoprotein with high molecular weight; Involved in lubrication and protection in normal tissues.	Normally expressed in the surface of epithelial cells; Highly expressed in various tumors, including multiple myeloma, breast cancer, and lung cancer, and associated with poor prognosis.		MUC1 is highly expressed in lung cancer and regulates the stemness and resistance via NF-κB and MAPK pathways.		MUC1-ADC
**PSCA**	A cell surface antigen that belongs to the Ly-6/Thy-1 family of glycosylphosphatidylinositol-anchored proteins; Unclear biological functions in normal tissue.	Mainly expressed in basal and secretory cells of the normal prostate; Highly expressed in over 80% of patients with prostate cancer.		PSCA is highly expressed in NSCLC and PSCA knockdown resulted in the inhibition of lung cancer cell growth.		PSCA-ADC
**PD-L1**	A significant and classical checkpoint in tumor immunotherapy; Combined with PD-1 to inhibit T cell activation and function for immune escape.	Widely expressed in immune cells and tumor cells, including NSCLC, melanoma cancer, and lymphoma.		PD-L1 is a significant clinical target for lung cancer, both SCLC and NSCLC.		Atezolizumab, Durvalumab
**B7-H3**	A transmembrane protein belongs to the B7 immunoglobulin family; An immunosuppressive molecule inhibiting T cell activation, proliferation, and cytokine secretion.	Rarely expressed in normal tissues, but highly expressed in primary malignant tumors; A valuable biomarker and target for solid tumor immunotherapy.		B7-H3 is highly expressed in lung cancer and is associated with poor prognosis and disease progression.		blocking mAbs, Fc-enhanced mAbs, ADCs
**DLL3**	A single-pass transmembrane protein and a part of the inhibitory NOTCH signaling pathway; Combined with NOTCH proteins to promote SCLC cell proliferation, invasion, and tumor progression.	Rarely expressed in normal tissues, but highly expressed in SCLC and neuroendocrine carcinoma.		DLL3 is an ideal target for SCLC treatment. The knockout of DLL3 decreased the SCLC cells proliferation while the overexpression increased their proliferation.		DLL3-ADC, BiTE

### Epidermal growth factor receptor (EGFR)

EGFR is a typical human epidermal growth factor receptor and is responsible for extracellular signal transduction into cells [59]. EGFR is expressed mainly on the cytomembranes of epithelial cells and several epithelium-derived tumors [60]. Approximately 60% of patients with NSCLC have EGFR mutations, and EGFR is a major therapeutic target for NSCLC [61]. A series of targeted drugs have been approved for clinical treatment, including gefitinib, afatinib, and osimertinib [62–64]. Moreover, based on its substantially elevated expression level in lung carcinoma cells, EGFR is a promising therapeutic target for CAR-T therapy [65]. In a clinical trial (NCT01869166) involving patients with chemotherapy-refractory or relapsed EGFR-positive (> 50%) NSCLC, EGFR CAR-T (coupled with 4-1BB and CD3ζ domains) cells presented EGFR-specific antitumor cytotoxicity and no unacceptable toxicity [65]. Another clinical trial (NCT03182816) revealed that nonviral piggyBac transposon system engineered EGFR CAR-T cell therapy is feasible and safe for the treatment of EGFR-positive advanced relapsed/refractory NSCLC [66]. Moreover, two clinical studies (NCT05060796 and NCT04153799) focused on the safety and efficacy of EGFR CAR-T modified by C-X-C Chemokine receptor type 5 (CXCR5) in patients with advanced-stage adult NSCLC, based on one preclinical research showing that the CXCR5 protein is capable of increasing T cell infiltration in tumors via the CXCR5-CXCL13 axis. Recently, the chemokine receptors CCR6 and PD-1 blocking scFv E27 were fused onto EGFR CAR-T and showed enhanced anti-tumor efficacy in a NSCLC xenograft model [67]. As a typical negative immune regulator, TGF-β is enriched in the TME of NSCLC and inhibits T cell proliferation and activity. To relieve this suppression, CAR-T cells were reconstructed to overexpress the mothers against decapentaplegic homologue 7 (SMAD7), a negative regulator of TGF-β downstream signaling. The overexpression of SMAD7 improved CAR-T cell persistence and performance [68]. The above studies suggest that EGFR CAR-T is a potential therapy for NSCLC and several combination strategies enable to improve its efficacy.

### Carcinoembryonic antigen (CEA)

CEA is an acidic glycoprotein with human embryonic antigen properties and it is highly expressed in the colon during fetal development. However, in certain digestive system neoplasms, including those of colorectal cancer, lung cancer, gastric carcinoma, and pancreatic cancer, the expression of CEA was upregulated [69]. According to several preclinical studies, the occurrence of brain metastases in patients with advanced-stage is closely associated with the serum concentrations of CEA [70]. Moreover, CEA is hardly expressed in other normal cells, except for at low levels in intestinal epithelial cells. Therefore, CEA is considered a promising therapeutic target for lung cancer and other solid tumors. CEA CAR-T has been tested in several clinical trials involving patients with solid tumor and metastases, including two phase I trials for lung carcinoma (NCT02349724 and NCT04348643). Moreover, some preclinical studies have focused on improving CEA CAR-T therapy. The knockout of E3 ubiquitin ligase Cbl-b via CRISPR system inhibited CEA CAR-T cell exhaustion and increased the secretion of IFN-γ and TNF-α, ultimately improving CEA + tumor cytotoxicity [71]. A recent study screened four reported humanized anti-CEA antibodies and individually inserted them into a third generation CAR structure followed by phenotype detection and function verification, which demonstrated that scFvs derived from different antibodies differ in cell proliferation, cytokine secretion, and anti-tumor characteristics [72]. These studies provide a theoretical basis and guide for optimizing CEA CAR-T therapy for the treatment of patients with lung cancer and other CEA + solid tumors.

### Mesothelin (MSLN)

As a glycosylated phosphatidylinositol-anchored protein, MSLN is rarely expressed in normal tissues, except for at low levels in bronchus, fallopian tube, placenta, and tonsil. High expression levels have been detected in tumor tissues, including mesothelioma, pancreatic cancer, and lung cancer [73, 74]. The abnormal high expression of MSLN has been shown to promote tumor cell proliferation, local invasion, and malignant metastasis [75]. Therefore, MSLN is considered a significant diagnostic and therapeutic target, and a suitable TAA for CAR-T therapy for the treatment of solid tumors. Most MSLN CAR-T clinical trials are ongoing involving patients with NSCLC. Nanoantibodies targeting PD-1 and CTLA-4 were fused onto CAR-T cells (NCT04489862 and NCT06248697) to improve their clinical efficacy. In one clinical trial (NCT03054298), low-dose MSLN CAR-T cells showed safety and feasibility regardless of receiving pretreatment with cyclophosphamide. However, severe pulmonary toxicity occurred in patients undergoing refusion with high-dose CAR-T cells. Therefore, it is critical to consider and detect the dynamic expression of MSLN in benign lungs before applying MSLN CAR-T cell therapy. To control potential side effects and enhance safety, a suicide element, iCasp9, was added to the CAR structure in another trial (NCT02414269) [76]. In addition, an HLA-gated safety device was assembled and added to CAR-T cells to confer specific antitumor cytotoxicity and protect normal MSLN-positive cells to eliminate the risk of severe inflammation [77]. Moreover, the coexpression of the cell chemokine receptors CCR2b and CCR4 improved MSLN CAR-T performance by enhancing migration and infiltration into lung cancer tissue in a mouse model [78].

### Human epidermal growth factor receptor 2 (HER2)

HER2 is a tyrosine kinase receptor belonging to the ERBB/HER family, and its major function is transferring and activating signals, similar to EGFR [79]. It is involved in cell proliferation and angiogenesis, but its extreme activation frequently implies the deterioration of epithelial cell cancers. Although the gene abnormality ratio of HER2 is 2–4% in lung cancers, which is lower than that of EGFR, HER2 is considered a promising biomarker and target for clinical diagnosis and treatment [80–82]. HER2 CAR-T therapy has been applied for the treatment of breast cancer, gastric carcinoma, sarcoma, and other malignancies [83–85]. For the treatment of lung cancer, the applications of HER2 CAR-T cells have focused mainly on preclinical studies. It has been reported that docetaxel, a chemotherapeutic agent, can induce HER2 CAR-T cell recruitment to the TME and enhance its anti-tumor effect in a mouse model of NSCLC [86]. Subsequently, high mobility group protein B1 (HMGB1) was confirmed to decrease the surface localization of PD-1 and promote its degradation, leading to an advanced tumor killability when combined with HER2 CAR-T cells [87]. These studies provide a theoretical foundation for improving immunotherapy efficacy. Thus, further exploration and clinical trials are essential for assessing the safety, feasibility, and efficacy of applying HER2 CAR-T for the treatment of lung cancer.

### Receptor tyrosine kinase-like orphan receptor 1 (ROR1)

ROR1 was first identified in 1992, and it plays essential roles in embryogenesis [88]. Recently, the ROR1 protein was found to be highly expressed in certain human neoplasms, including chronic lymphocytic leukemia (CLL), breast cancer, ovarian cancer, and lung cancer [88]. In fact, an increasing number of studies have identified that ROR1 as a potential target for the treatment of malignancies. A humanized ROR1 monoclonal antibody (mAb), cirmtuzumab has been used for CLL treatment and has achieved effective clinical outcomes (NCT02222688) [89]. In NSCLC cell lines, silencing ROR1 led to inhibited activity of the PI3K/AKT/mTOR signaling pathway, which ultimately induced apoptosis and resulted in antiproliferative effects [90]. Several CAR-T therapies targeting ROR1 with different antigen recognition domains have been evaluated in solid tumor xenografted mouse models. Combination therapy with cyclophosphamide/oxaliplatin and anti-PD-L1 antibodies synergistically improved ROR1 CAR-T cell-mediated lung cancer control and prolonged survival time, providing a strategy to improve the effect of ROR1 CAR-T cell effect in the clinic [91]. In another study, the pressure in the TME was shown to blunt the efficacy of ROR1 CAR-T cell therapy against lung cancer cell line A549 by upregulating PD-L1 expression [92]. These studies suggest that checkpoints cannot be ignored during ROR1 CAR-T therapy. The first clinical trial of ROR1 CAR-T therapy revealed tumor regression in four patients with B cell malignancies, but the risk of death was also detected (NCT05588440). Another trial recruiting patients with stage IV NSCLC or triple negative breast cancer (TNBC) evaluated the efficacy and the toxicity of ROR1 CAR-T with different refusion doses (NCT02706392) [91]. More studies on ROR1 CAR-T cells are needed.

### Mucin 1 (MUC1)

MUC1 is a transmembrane mucin that is gradually distributed on the normal mucosal surface of tissues [93]. Extensive glycosylation (O-GalNAc glycosylation and N- GalNAc glycosylation) promotes its biological function and stability [94]. However, the overexpression and abnormal activation of MUC1 facilitate tumor cell invasion and vascularization, including in multiple myeloma, breast cancer, and lung cancer [93]. Based on its significantly high expression in malignant tissues, MUC1 has been regarded as a potential target for drug development, including antibody-drug conjugates (ADCs), vaccines, and MUC1 CAR-T [95]. MUC1 CAR-T cells have been verified to efficiently eliminate MUC1-positive malignant tissues, including T cell leukemia, pancreatic cancer, NSCLC, breast cancer, and pancreatic ductal adenocarcinoma in preclinical xenograft models [96]. Moreover, an anti-PD-1 antibody enhances the anti-tumor ability of tandemly targeted MUC1 and PSCA CAR-T cells against NSCLC [97]. In a clinical trial using allogeneic MUC1 CAR-T cells to target advanced or metastatic epithelial derived solid tumors (NCT05239143), a patient with breast cancer and two patients with gastroenteric tumor achieved partial remission and stable conditions. Other clinical trials recruiting patients with lung cancer are ongoing to test and assess the safety and preliminary efficacy of MUC1 CAR-T (NCT02587689, NCT03356808, and NCT04025216). Another clinical study combined MUC1 CAR-T with PD-1 knockout to treat patients with advanced NSCLC, might create superior malignant tumors elimination capability (NCT03525782).

### Prostate stem cell antigen (PSCA)

PSCA is a member of the Thy-1/Ly-6 family of glycosylphosphatidylinositol-anchored cell surface antigens [98]. It was originally discovered to be expressed mainly in basal and secretory cells of the normal prostate. And over 80% of patients with prostate cancer exhibit PSCA overexpression [99]. High PSCA expression has subsequently been reported in several other malignancies, including bladder, pancreatic, and renal cancers. In NSCLC, the vast majority of clinical lung cancer tissues have high PSCA protein expression compared with that in normal lung epithelium, and PSCA knockdown resulted in the inhibition of lung cancer cell growth, which suggests that PSCA may be a functionally important target for the treatment of NSCLC [100]. PSCA CAR-T has initially presented anti-tumor activity against pancreatic cancer [101]. Then, PSCA CAR-T demonstrated robust anti-tumor cytotoxicity against gastric cancer and lung cancer. The combined targeting of PSCA and MUC1 further enhances the antitumor efficacy of CAR-T cells in tumor models of NSCLC [102]. A clinical study of multiple antigens targeted CAR-T for the treatment of lung cancer is under recruiting, including PSCA CAR-T (NCT03198052). Actually, the clinical efficacy, safety, and feasibility of PSCA CAR-T for the treatment of lung cancer need to be further assessed.

### Programmed death-ligand 1 (PD-L1)

PD-1/PD-L1 has been regarded as a pair of significant and classical checkpoints in tumor immunotherapies [103]. Major tumor cells overexpress PD-L1 to achieve immune tolerance and inhibit T cell activation and function [104]. PD-L1 antibodies have demonstrated remarkable results in both preclinical and clinical studies. In a recently completed clinical trial (NCT02117167) involving over 1000 patients with NSCLC, the PD-L1 mAb, atezolizumab obviously increased overall survival time, even for patients with PD-L1 negative NSCLC [105]. In addition to PD-L1 antibody treatment, PD-L1 CAR-T has been developed, in which the scFv was derived from atezolizumab. PD-L1 CAR-T could efficiently lyse PD-L1 + tumor cells and suppress the growth in an NSCLC tumor model [106]. Furthermore, the combination of the PD-L1 CAR structure with other antigen targeted CAR structures could enhance antitumor efficacy and reduce the risk of on-target off-tumor toxicity in the treatment of PD-L1 + solid tumors [107]. PD-L1 CAR-T has been used in a clinical trial (NCT03330834) for the treatment of advanced lung cancer. Only one patient was recruited and serious cytokine release syndrome occurred after CAR-T refusion. Another clinical trial (NCT03060343) involved patients with PD-L1 positive and recurrent or refractory NSCLC. Collectively, PD-L1 CAR-T has shown promising preclinical results, but further studies and clinical trials are essential for its clinical application for lung cancer and other solid tumors.

### B7-H3

B7-H3, also called CD276, belongs to the B7 immunoglobulin family. Recent studies have revealed that B7-H3 enabled to inhibit T cell activation, proliferation, and cytokine secretion, as an immunosuppressive molecule [108]. B7-H3 is rarely expressed in normal tissues, but it is highly expressed in primary malignant tumors. Given its expression level bias, B7-H3 has been considered a valuable biomarker and target for solid tumor immunotherapy [109, 110]. Multiple B7-H3 based treatment strategies have been implemented and assessed, including blocking mAbs, Fc-enhanced mAbs, ADCs, and B7-H3/IL-15 TriKE [111, 112]. Notably, B7-H3 expression level is elevated in patients with SCLC and NSCLC. One recent clinical trial demonstrated that targeting B7-H3 ADC drug (ifinatamab deruxtecan) significantly improved the response rates and prolonged the survival time of patients with lung cancer, which makes B7-H3 an ideal target for lung cancer treatment [113]. B7-H3 CAR-T has exhibited favorable cytotoxicity against solid tumors, such as breast cancer, glioblastoma, prostate cancer, osteosarcoma, and lung cancer [114]. Two clinical trials of the application of B7-H3 CAR-T for the treatment of lung cancer are ongoing (NCT03198052 and NCT04864821). Moreover, in in vivo xenotransplant models of orthotopic and metastatic NSCLC, the co-expression of cell chemokine receptor CCR2b markedly improved the ability of B7-H3 CAR-T cells to cross the blood-brain barrier and enhanced their potential efficacy against brain metastases [115]. The heterogeneous and immunosuppressive TME suppressed CAR-T cell therapy in solid tumors. Nanozymes have merits in modulating the immunosuppression of TME and applying them in combination effectively improved the therapeutic index of B7-H3 CAR-T cells for the treatment of NSCLC in preclinical studies by increasing activation and infiltration [116]. These studies suggested that B7-H3 CAR-T has great potential for lung cancer immunotherapy.

### Delta-like ligand 3 (DLL3)

DLL3 is a single-pass transmembrane protein and a part of the inhibitory NOTCH signaling pathway. DLL3 was robustly expressed in SCLC cells across disease stages and lines of therapy according to an international real-world study [117]. Recent preclinical studies showed that the knockout of DLL3 decreased the SCLC cell proliferation while the overexpression increased their proliferation [118]. The high-expressing of DLL3 suppressed immune-related pathways and dendritic cell function which inhibited the immunotherapy effect of checkpoint inhibitors. These studies suggested that DLL3 was a potential therapeutic target for SCLC [119]. And CAR-T cells targeting DLL3 have been used for the treatment of SCLC. In a preclinical study, allogeneic DLL3 CAR-T cells presented enough efficacy and safety [58, 120]. Meanwhile, three clinical trials about DLL3 CAR-T therapy for patients with SCLC have been initiated (NCT06348797, NCT05680922, and NCT06384482). Collectively, with the rapid advancement of more preclinical studies and clinical trials, DLL3-targeted CAR-T has been expected to provide a hopeful and efficient treatment option for patients with SCLC. The clinical trials for CAR-T cell therapy targeting these antigens can be seen in Table 2.

**Table 2:** The clinical studies of CAR-T therapy in lung cancer

Antigen targets	Clinical trial ID	Patient type	Phase	Status	Characteristics & Result
**EGFR**	NCT01869166	Patients with EGFR positive advanced/unresectable operation solid tumors	Phase I/II	Unknown	Eleven patients with EGFR positive (>50% expression), relapsed/refractory NSCLC received escalating doses of EGFR CAR-T cell infusions. Two patients obtained partial response and five had stable disease for two to eight months. Pathological eradication of EGFR positive tumor cells was observed in tumor biopsies after CAR-T cell treatment.
	NCT03182816	Patients with EGFR positive advanced relapsed/refractory NSCLC	Phase I/II	Unknown	Nine patients with advanced relapsed/refractory EGFR positive NSCLC received two cycles of the piggyBac-generated EGFR CAR-T cells. One patient showed a partial response and lasted for more than one year, while six had stable disease, and two had progressed disease. This clinical trial revealed that the non-viral piggyBac transposon system-engineered EGFR CAR-T cell therapy is feasible and safe in treatment of EGFR positive NSCLC patients.
	NCT05060796	Patients with advanced adult NSCLC	Early Phase I	Recruiting	This trial is a single arm, open label, Phase I study to evaluate the safety and efficacy of CXCR5 modified EGFR CAR-T Cells in EGFR positive patients with advanced NSCLC.
	NCT04153799	Patients with advanced adult NSCLC	Phase I	Unknown	This trial is a single arm, open label, Phase I study to evaluate the safety and efficacy of CXCR5 modified EGFR CAR-T Cells in EGFR positive patients with advanced NSCLC.
**CEA**	NCT02349724	Patients with CEA positive gastric cancer, lung cancer, pancreatic cancer, breast cancer, and colorectal cancer	Phase I	Unknown	Ten patients with CEA positive colorectal cancer were first recruited for CAR-T cell therapy. Two patients showed tumor shrinkage and several patients obtained stable disease after treatment with CEA CAR-T cells, without significant CAR-related toxicity. It suggested the safety and efficacy of CEA CAR-T therapy for solid tumors, including lung cancer.
	NCT04348643	Patients with relapsed and refractory CEA positive cancer	Phase I/II	Unknown	This trial is a single arm study to evaluate the efficacy and safety of CEA CAR-T cell therapy for patients with CEA positive solid tumors and obtain the recommended dose and infusion plan.
**MSLN**	NCT04489862	Patients with MSLN positive advanced solid tumors	Early Phase I	Unknown	This trial is a single arm, open label, dose escalation clinical study to evaluate the safety, efficacy, and tolerability of autologous MSLN CAR-T cells with PD-1 nanoantibody secreting for MSLN positive solid tumors.
	NCT06248697	Patients with MSLN positive advanced solid tumors	Early Phase I	Recruiting	This trial is a single arm, open label, dose escalation clinical study to evaluate the safety and tolerability of autologous MSLN CAR-T cells with PD-1 and CTLA-4 nanoantibodies secreting for MSLN positive solid tumors.
	NCT03054298	Patients with MSLN expressing cancers	Phase I	Completed	This trial was aimed to establish the safety and feasibility of both intravenous administration and local delivery of MSLN CAR-T cells with or without lymphodepletion. Total 30 patients were consented and enrollmented. Normal dose showed enough safety, while high dose showed pulmonary toxicity.
	NCT02414269	Patients with malignant pleural disease from mesothelioma, lung cancer, or breast cancer	Phase I/II	Active	The purpose of this Phase I study is to test the safety of different doses of specially prepared T cells collected from blood. 21 patients with malignant pleural disease were treated with CAR-T cells and the results showed no evidence of on-target, off-tumor or therapy related toxicity.
**HER2**	NCT03198052	Patients with HER2 positive cancers	Phase I	Recruiting	In this Phase I study, the safety, tolerance, and preliminary efficacy of CAR-T cells were evaluated and tested, targeting multiple solid tumors, including HER2.
**ROR1**	NCT02706392	Patients with Advanced ROR1 positive cancers	Phase I	Terminated	This Phase I trial studies the best dose and side effects of CAR-T cells in treating patients with ROR1 positive tumors. Four TNBC patients have been enrolled, treated, and are evaluable for response. It suggested that ROR1 CAR-T cells can be safely transferred, expand in vivo in patients.
**MUC1**	NCT02587689	Patients with MUC1 positive advanced refractory solid tumors	Phase I/II	Unknown	The purpose of this trial is to determine whether autologous CAR-T cells recognizing MUC1 is safe and effective for patients with relapsed or refractory solid tumor.
	NCT03356808	Patients with lung cancers	Phase I/II	Unknown	The purpose of this trial is to assess the feasibility, safety, and efficacy of CAR-T cells targeting lung cancer and the cancer targeting antigens include known tumor antigens, such as MUC1, MAGE-A1, MSLN, as well as novel cancer antigens. And another purpose is to learn more about the persistence and function of CAR-T cells in body.
	NCT04025216	Patients with advanced MUC1 positive solid tumors and multiple myeloma	Phase I	Terminated	In this Phase I study, the safety, tolerability, feasibility, and preliminary efficacy of CAR-T cells recognizing MUC1 were assessed for patients with MUC1 positive solid tumors and multiple myeloma.
	NCT03525782	Patients with advanced adult NSCLC	Phase I/II	Unknown	The study is to assess the safety and efficacy of the MUC1 CAR-T cells with PD-1 knockout for patients with advanced NSCLC. 20 participants received at least one cycle of MUC1 CAR-T cell treatment, in which 11 patients presented with stable disease while 9 had progressive disease. The result suggests that the treatment with PD-1 disrupted MUC1 CAR-T cell is safe and well tolerated by all NSCLC patients.
**PSCA**	NCT03198052	Patients with PSCA positive cancers	Phase I	Recruiting	In this Phase I study, the safety, tolerance, and preliminary efficacy of CAR-T cells were evaluated and tested, targeting multiple solid tumors, including PSCA.
**PD-L1**	NCT03330834	Patients with advanced lung cancer after standard treatment failure	Phase I	Terminated	The purpose of this trial is to assess the safety and toxicity of CAR-T cell immunotherapy targeting PD-L1 in patients with advanced lung cancer after standard treatment failure. Only one patient was recruited and serious cytokine release syndrome occurred after CAR-T refusion.
	NCT03060343	Patients with recurrent or refractory NSCLC	Early Phase I	Unknown	The purpose of this trial is to determine the safety and feasibility of CAR-T cells targeting PD-L1 and CD80/CD86 in patients with relapsed or refractory NSCLC.
**B7-H3**	NCT03198052	Patients with B7-H3 positive cancers	Phase I	Recruiting	In this Phase I study, the safety, tolerance, and preliminary efficacy of CAR-T cells were evaluated and tested, targeting multiple solid tumors, including B7-H3.
	NCT04864821	Patients with B7-H3 positive advanced solid tumors	Early Phase I	Unknown	In this clinical experiment, the sponsors aim to explore the best model of B7-H3 CAR-T therapy for solid tumor by intravenous and local tumor injection, which will bring new hope to patients with osteosarcoma, neuroblastoma, gastric cancer, and lung cancer.
**DLL3**	NCT06348797	Patients with relapsed/refractory SCLC	Phase I	Not yet recruiting	The purpose of this trial is to evaluate the safety and feasibility of DLL3 CAR-T cells with PD-L1 and 4-1BB antibodies in patients with relapsed or refractory SCLC. And 16 patients are planned to enroll in dose expansion phase who was assign two groups with/without bridge radiotherapy.
	NCT05680922	Patients with extensive stage SCLC	Phase I	Recruiting	This is a phase I, first-in-human, open label, multicenter, dose escalation, and expansion study of DLL3 targeted CAR-T cells in subjects with extensive stage SCLC or large cell neuroendocrine lung cancer. Up to 41 subjects would be treated in this study. The objectives of the study include characterization of safety and tolerability, evaluation of the recommended phase II dose, and assessment of preliminary antitumor activity.
	NCT06384482	Patients with recurrent/​refractory SCLC and lung large cell neuroendocrine carcinoma	Phase I	Recruiting	This study is a FIH dose escalation clinical study, with single arm, open label and design, in order to observe the preliminary safety and pharmacokinetic of DLL3 CAR-T cells in patients with recurrent/refractory SCLC and lung large cell neuroendocrine carcinoma. The study will enroll at most 35 participants.

### Other antigen targets

To increase the efficacy and extend the application of CAR-T therapy for the treatment of lung cancer, many studies have been performed to excavate potential new antigen targets, including the disialoganglioside GD2, the tyrosine kinase receptor EphA2, the melanoma-associated antigen MAGE-A1, and the protein tyrosine kinase 7 (PTK7) [121–124]. Further clinical trials for evaluating the security and efficacy of these new targets are needed.

## Strategies to enhance CAR-T efficacy for the treatment of lung cancer

**Fig. 2:**
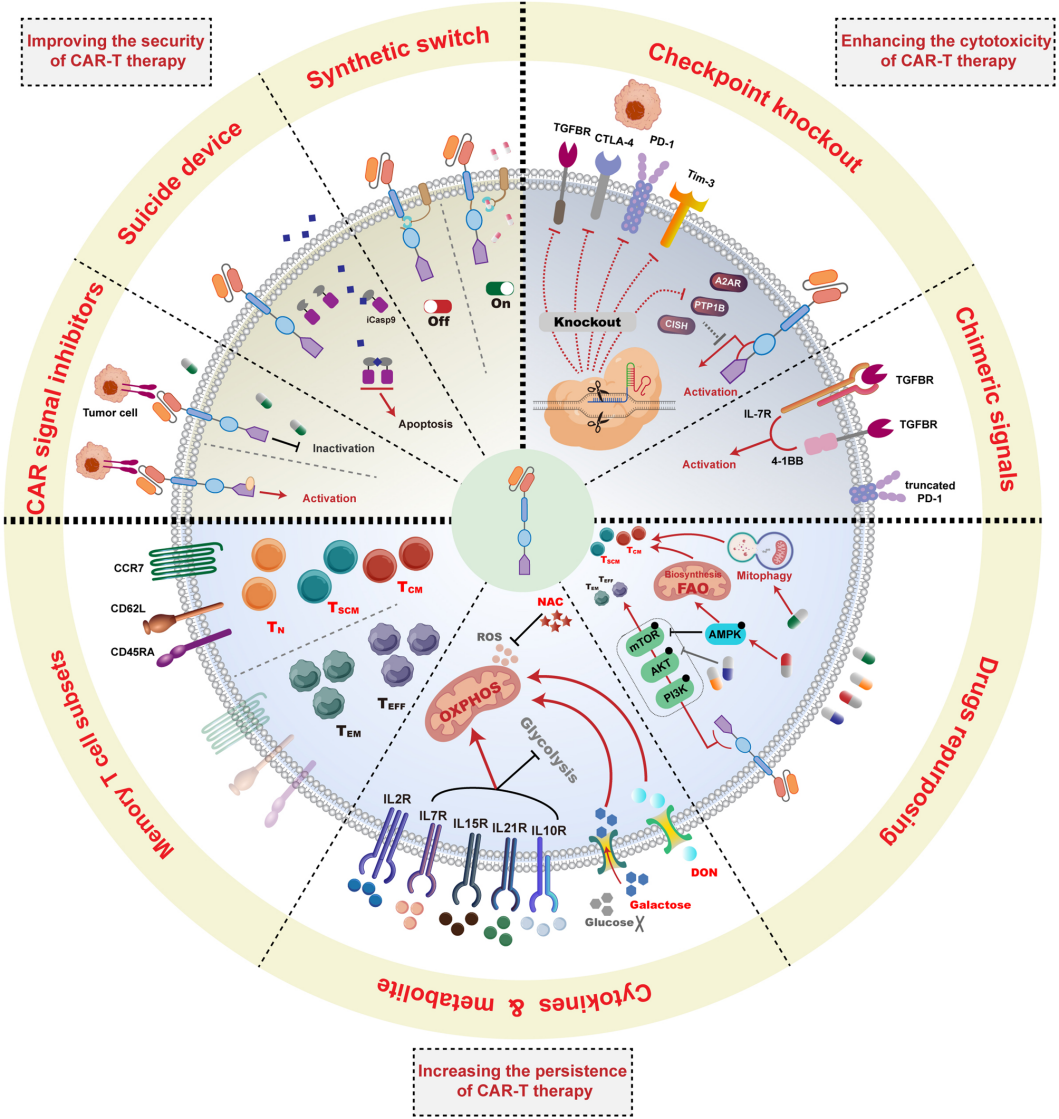
The schema of strategies to enhance CAR-T efficacy for lung cancer, including improving the security of CAR-T therapy, enhancing the cytotoxicity of CAR-T therapy, and increasing the persistence of CAR-T therapy

Although CAR-T therapy has achieved great success in treating hematological malignancies, several bottlenecks and challenges still need to be resolved for its application in solid tumors including lung cancer. In this section, we describe and discuss several different approaches and strategies that have been recently adopted in vitro and in vivo to improve the efficacy of CAR-T cell therapy and overcome existing barriers, including on-target off-tumor toxicity (OTOTT), cytokine release syndrome (CRS), impaired CAR-T cell function, limited persistence and infiltration, and immunosuppressive TMEs (Fig. 2).

### Improving the security of CAR-T therapy

Security is an invariable core of CAR-T therapy and other clinical immunotherapies. OTOTT and CRS are two major risks that threaten the security of CAR-T therapy [125, 126]. OTOTT begins with CAR-T cells induced recognition and combination of non-malignant tissues expressing the antigen targets. The antigen could activate CAR-T cells and trigger effector functions, including the release of granzymes and perforin and the secretion of cytokines, which resulted in tissue or organ destruction. OTOTT has been observed in several clinical trials involving patients with solid tumor. In a phase I clinical trial (NCT01212887), the application of carcinoembryonic antigen related cell adhesion molecule 5 (CEACAM5) CAR-T cells for patients with CEACAM5 + malignancy tumor resulted in severe adverse manifestations, including tachypnoea, pulmonary infiltrates and respiratory distress. Subsequent research revealed CEACAM5 expression in non-malignant lung resection samples was more than that in 60% of patients in the trial. In another clinical trial of EGFR CAR-T for the treatment of advanced-stage biliary tract cancer and metastatic pancreatic cancer (NCT01869166), severe mucosal and cutaneous adverse events were observed. Collectively, these studies showed the likelihood of severe OTOTT in patients with solid tumors receiving CAR-T cells treatment. Moreover, CRS is another severe adverse event. In the early stage of treatment, CAR-T cells might be over-active and violently proliferate, accompanied by the release of abundant cytokines [127]. These cytokines further activated immune cells via positive feedback and caused a systemic inflammatory response and severe damages to tissues or organs [127]. Therefore, these toxicities observed in clinical trials of CAR-T therapy have encouraged the development of technologies to improve the security of CAR-T therapy while maintaining the anti-tumor efficacy.

Although increasing potential antigen targets for lung cancer have been excavated (shown in Sect. 3), the majority of these targets are expressed at low levels in non-malignant tissues and the risk of OTOTT remains. Therefore, superior antigen targets still need to be identified. Moreover, a series of ingenious and hopeful strategies to improve the security of CAR-T therapy have been explored. Dasatinib, a clinical tyrosine kinase inhibitor, can inhibit CAR signal and CAR-T cell activation, and the administering dasatinib as an adjuvant reduced the incidence of CRS in a lymphoma mouse model by inhibiting CAR-T cell differentiation and exhaustion [128]. Several clinical trials of CD19/BCMA-targeted CAR-T cells combined with dasatinib are ongoing (NCT04603872 and NCT05523661). Suicide device has been also introduced into CAR structure via the fusion of a modified FK-binding protein and Caspase 9, which could efficiently eliminate over 90% of CAR-T cells to prevent CRS and other side-effects [129, 130]. In addition, synthetic biology has provided novel solutions. A resveratrol-responsive trans-activator and a trans-repressor were designed to engineer a controllable device in which the CAR-T cell function and anti-tumor effects were regulated in a resveratrol-titratable manner [131]. Recently, a protease-based CAR platform was shown to demonstrate that CAR-T activity could be regulated via an FDA-approved small molecule and it manifested full functional capacity with the molecule and no leaky activity in the absence of the molecule [132]. Moreover, our recent work engineered a controllable and reversible switch for CAR-T and CAR-NK cell immunotherapies via a genetic code expansion system [133]. Collectively, these logic-gated and switch-receptor strategies enable the flexible and spatial control of CAR-T cell function with fewer adverse effects. However, these devices have not yet been tested clinically in the context of CAR-T therapy, and further verification in clinical trials is needed.

Moreover, some clues have revealed that the CAR structure is capable of affecting the toxic effects of CAR-T therapy. Genetically altering sequences of the extracellular and intracellular domains of the CD8α molecule resulted in reduced levels of cytokine release in CD19 CAR-T cells [134, 135]. In a subsequent clinical trial, no obvious CRS was observed in patients with B cell lymphoma after receiving the modified CAR-T cells treatment (NCT02842138) [136]. The traditional scFv originated from mouse antibodies and it might induce immune responses by the host immune cells. The use of humanized antibody fragments for the CAR structure enabled to reduce the immunogenicity and decrease the risk of CRS [137]. In conclusion, seeking for superior antigen targets, flexibly controlling CAR-T function, and reconstructing the CAR structures are three crucial strategies for improving the security and reducing the side-effects of CAR-T therapy.

### Enhancing the cytotoxicity of CAR-T therapy

The immune checkpoint is a critical path through which tumor cells evade the immune system, especially cells from solid tumors. To evade the immune checkpoint signals, genetic and pharmacological inhibition strategies have been adopted in CAR-T therapy. The PD-1/PD-L1 axis was the most famous pair of immune checkpoint molecules, for which multiple targeted drugs have been researched and developed [14]. The interaction between PD-L1 and PD-1 induces T cell disability and exhaustion. Similarly, the PD-1/PD-L1 axis also suppresses the anti-tumor effects of CAR-T cells [138]. Genetic knockout of PD-1 during the CAR-T preparation stage has demonstrated promising results for the treatment of different tumors [139, 140]. A recent clinical trial reported that PD-1 interference enhanced the anti-tumor immune functions of CD19 CAR-T cells when applied to patients with relapsed/refractory aggressive B cell non-Hodgkin lymphoma (NCT04213469) [141]. In addition, the CAR-T cells loaded with a PD-1-blocking scFv enabled to protect CAR-T cells from PD-1 inhibition and potentially avoid the toxicities associated with systemic checkpoint inhibition [142]. Both anti-PD-1 antibody and PD-1 genetic knockout have been verified to enhance the anti-tumor ability of CAR-T cells against NSCLC [97]. It has been substantiated that the knockout or inhibition of other immune checkpoints, including Tim-3, CTLA-4, and LAG-3, significantly enhanced the cytotoxic function, cytokine production and proliferation of multiple CAR-T cells [143–145]. However, further studies are needed to verify their effects on CAR-T cells targeting lung cancers.

Some known and novel negative immune regulators have gradually emerged as targets for improving the efficacy of CAR-T therapy [146]. Protein tyrosine phosphatase 1B (PTP1B) enable to attenuate cytokines-stimulated JAK/STAT signaling by dephosphorylating and deactivating JAK2. It is upregulated in tumor infiltrating T cells and limits the proliferation and cytotoxicity of T cells to contribute to tumor growth. Genetic deletion and pharmacologic inhibition of PTP1B enhanced the performance of HER2 CAR-T against solid tumors [147, 148]. Another JAK-STAT suppressor, cytokine-inducible SH2-containing protein (CISH), inhibits TCR and cytokine signaling in T and natural killer (NK) cells [149]. CAR-T cells with CISH deficiency exhibit increased cytokine secretion, enhanced durability, and superior anti-tumor activity [150]. As one of the key immunosuppressive metabolites in the TME of solid tumors, adenosine interacts with the adenosine A2A receptor (A2AR) to activate adenosine signaling, inhibiting the activation of multiple immune cells and suppressing the anti-tumor response [151]. Using CRISPR/Cas9 to knock out A2AR was found to enhance the anti-tumor effect of P4 CAR-T against lung cancer and of HER2 CAR-T cells against breast cancer [152]. TGF-β interacts with the TGF-β receptor (TGFBR) on the T cell surface, activating the downstream signaling [153]. Increasing evidence suggests that the TGF-β pathway might be a potential target for enhancing the efficacy of CAR-T therapy against solid tumors. Knocking out TGFBRII in CAR-T cells using the CRISPR-Cas9 system promoted the differentiation of CAR-T cells into central memory and effector cells, enhancing the cytotoxicity in solid tumors [154]. Moreover, another study designed a TGF-β/IL-7 chimeric switch receptor to convert TGF-β signaling into immune-activating IL-7 signaling. Compared with conventional CAR-T cells, CAR-T cells using this switch receptor exhibited significantly prolongs overall survival time [47]. A recent study demonstrated that CD38 is a potential biomarker for CAR-T cell exhaustion and that inhibiting CD38 activity reverses CAR-T cell exhaustion and enhances its cytotoxicity and anti-tumor response independent of the scFv or intercellular costimulatory domain [155, 156]. Mechanistically, the inhibition of CD38 suppresses the glycolysis via the CD38-NAD-SIRT1 axis. Collectively, these studies suggest that inhibiting immune checkpoints and negative regulators is a valuable strategy for enhancing the cytotoxicity of CAR-T therapy and has the potential for clinical applications.

### Increasing the persistence of CAR-T cells

Similar to traditional treatments, recurrence is an issue for CAR-T cell therapy, as approximately half of patients receiving CAR-T cells experience tumor recurrence [157]. The easy exhaustion and poor persistence of CAR-T cells are two vital factors, characterized by a gradual decrease in cytokine production and secretion, loss of response and cytotoxicity to tumor cells, slowed proliferation, and accelerated apoptosis. In this section, we discuss and summarize the latest strategies and methods used to improve CAR-T cells persistence and performance.

#### Different T cell subsets present diverse persistence performance

During in vitro preparation, T cells are powerfully activated by CD3 and CD28 signal stimulation, cytokine incubation, and transgenic CAR transduction, which results in CAR-T cells rapidly proliferating and differentiating from naïve state to the effector state. Defined by several surface markers (CD45RA, CD45RO, CD95, CCR7, CD62L, etc.), T cells are subdivided into five principal and typical subsets: naïve T cells (T_N_), stem cell memory T cells (T_SCM_), central memory T cells (T_CM_), effector memory T cells (T_EM_), and effector T cells (T_EFF_) [157]. Different subsets have diverse functional characteristics and performances. T_N_ cells highly express CD45RA, CD62L, and CCR7, and exhibit strong durability and proliferation. T_N_ cells could localize within lymph nodes [158]. However, the subset would be induced to be terminally differentiated during CAR-T cells preparation stage. T_SCM_ cells are located in the initial step of T_N_ cells differentiation after stimulation, with mighty self-renew ability and high expression of CD45RA, CD62L, CCR7, and CD95 [159]. T_SCM_ cells occupied approximately 2% of human peripheral blood mononuclear cell (PBMC) derived T cells [160, 161]. T_CM_ cells also originate as T_N_ cells that undergo antigen stimulation, with the expression of CD62L, CCR7, CD95, and the CD45RO isoform instead of the CD45RA isoform [157]. The long-term memory characteristic enables them to rapidly proliferate and effectively respond after secondary antigen exposure. T_EM_ and T_EFF_ cells lack the expression of CCR7 and CD62L, which dramatically reduces their homing ability. They could exhibit effective cytotoxicity after stimulation with tumor antigens. However, T_EFF_ cells inevitably lose their effector function and easily become exhausted. Among the five T cell subsets, T_SCM_ and T_CM_ cells exhibit outstanding self-renewal ability and long persistence [159]. Increasing the proportion of T_SCM_ and T_CM_ cell subsets may enhance the efficacy of CAR-T therapy, which is supported by recent studies [162, 163]. Two clinical trials revealed that central memory-derived CD19 CAR-T cells exhibited improvement in expansion for the treatment of relapsed intermediate-grade B cell non-Hodgkin lymphoma (NHL) (NCT01318317 and NCT01815749) [164, 165]. Another preclinical study produced CD19 CAR-T cells originating from CD62L + CD45RA + T_N_ and T_SCM_ cells, which performed superior antitumor activity and counteracted leukemia rechallenge in hematopoietic stem/precursor cell humanized mice, with increased expansion rates, enhanced memory phenotype, and decreased the risk of CRS [166]. Accordingly, compared with T_EFF_ and T_EM_ cells, T_N_, T_SCM_, and T_CM_ cells exhibited prolonged persistence and enhanced durability. Increasing the proportions of T_N_, T_SCM_, and T_CM_ cells is a potential strategy to enhance the efficacy of CAR-T therapy.

#### Manufacturing conditions influence CAR-T persistence

Different T cell subsets have different energy requirements. During in vitro manufacturing, metabolic reprogramming occurs at the same time as T cell activation and differentiation [167]. For T_N_ cells, the cellular metabolism is gentle and energy is gained mainly from OXPHOS and fatty acid oxidation (FAO). Similarly, the metabolic patterns of T_SCM_ and T_CM_ cells depend mainly on OXPHOS and FAO with low-grade glycolysis [168]. Once stimulated by CD3 and CD28 signaling, T_N_ cells accelerate their metabolism rate and energy production via aerobic glycolysis (Warburg effect) to meet the biosynthetic demands, and that results in the differentiation of T_N_ cells into T_EFF_ cells [169]. Given that metabolism patterns regulate the CAR-T cell differentiation and subset proportions, strategies to control CAR-T cell metabolism to increase the proportions of T_SCM_ and T_CM_ cells and increase persistence have been explored. In addition to IL-2, a series of cytokines, including IL-7, IL-15, IL-21, and IL-10, have been used during the preparation stage [170–173]. The application of these cytokines promotes CAR-T cells stemness by inhibiting glycolysis and promoting OXPHOS, further enhancing CAR-T cells function and antitumor immunity. Notably, the combination of IL-7 and IL-15 has been verified to enhance the antitumor activity of EGFR CAR-T against lung cancer. Moreover, a series of metabolites and intermediates have been demonstrated to modulate CAR-T cell performance. Short-chain fatty acids (SCFAs) promote mitochondrial oxidation through fatty acid catabolism, and the supplement could inhibit glycolytic reliance, enhancing the memory potential and anti-tumor efficacy of CAR-T cells [174]. Glucose is the conventional carbon source for adoptive cellular immunotherapy, but a recent study demonstrated that replacing the glucose in media with galactose for CAR-T cells can enhance the mitochondria metabolism [175]. In addition to glycometabolism, glutamine metabolism is another target for modulating CAR-T cell differentiation [176]. Glutamine inhibition by the addition of glutamine antagonist 6-diazo-5-oxo-L-norleucine (DON) into the culture endows CAR-T cells with improved mitochondrial OXPHOS using fatty acids, reduced glycolytic activity, and increased ratio of T_N_ and T_CM_ subsets, resulting in more robust elimination ability of tumor burdens [176]. N-acetylcysteine (NAC), a well-known broad antioxidant that functions by providing precursors for glutathione biosynthesis and scavenging ROS, has been verified to drive self-renewing memory CD8 + T cells and enhance the antitumor immunity when applied alongside with CAR-T cells [177].

#### Drug repurposing to promote CAR-T persistence

Given that metabolism is a significant determinant of CAR-T cell differentiation and the therapeutic efficacy, several approved drugs targeting metabolic pathways or other diseases have been applied as novel adjuvants to CAR-T therapy. The PI3K/AKT/mTOR pathway is a bridge linking TCR signaling and T cell metabolism, and its activation could accelerate glycolysis and effector cell differentiation [178, 179]. Inhibiting PI3K/AKT/mTOR via a series of inhibitors, including LY294002, idelalisib, duvelisib, ibrutinib, and rapamycin, has been verified to modulate the formation of memory T cells and enhance the long persistence of CAR-T cells [180, 181]. Moreover, epigenetic modifications shape the differentiation trajectory and regulate the metabolism and function of CAR-T cells. A serendipitous case revealed that disruption of tet methylcytidine dioxygenase 2 (TET2) promotes the therapeutic efficacy of CAR-T cells [182]. Abundant subsequent studies have focused on the epigenetic regulation in CAR-T therapy. Decitabine, a DNA methylation inhibitor approved for clinical use, was proven to modify the exhaustion-associated DNA methylation program in T cells. CAR-T cells treated with decitabine at a low-dose could eradicate bulky tumors and establish effective recall responses upon tumor rechallenge by increasing the expression of proliferation and memory associated proteins [183]. Recently, a high-throughput screening of chromatin modifying drugs revealed that the class I histone deacetylase inhibitors (HDACis), M344 and chidamide could enhance CAR-T cell anti-tumor efficacy by increasing resistance to exhaustion via the mechanistic decrease in HDAC1 expression and increase in H3K27ac activity [184]. Metformin, a drug that has been widely used to treat type II diabetes for approximately 60 years, has been well studied for its ability to inhibit tumor growth. Metformin treatment could increase the ratios of T_SCM_ and T_CM_ cells in HER2 CAR-T cells by regulating the AMPK/miR-107/Eomes/PD-1 pathway [185, 186]. Interestingly, some researches have applied natural products alongside CAR-T cell therapy. Urolithin A (UA) is a natural metabolite obtained from pomegranates. UA supplementation induces mitophagy and inhibits the progression of ageing-related diseases. UA-induced mitophagy was reported to promote the expansion of T_SCM_ cells and strongly induced a favorable anti-tumor immunity of CAR-T cells [187, 188]. Our previous study demonstrated that a traditional lung cancer target drug, afatinib enables to optimize the anti-tumor efficacy of CAR-T cells [189]. Collectively, these studies revealed that the application of several traditional drugs alongside CAR-T therapy could present unexpected effects on persistence and anti-tumor cytotoxicity, providing new approaches for the optimization of cellular immunotherapies. Moreover, some of these drugs have already been approved and could quickly be applied in clinical trials.

## Other cell-based immunotherapies in lung cancer

**Fig. 3:**
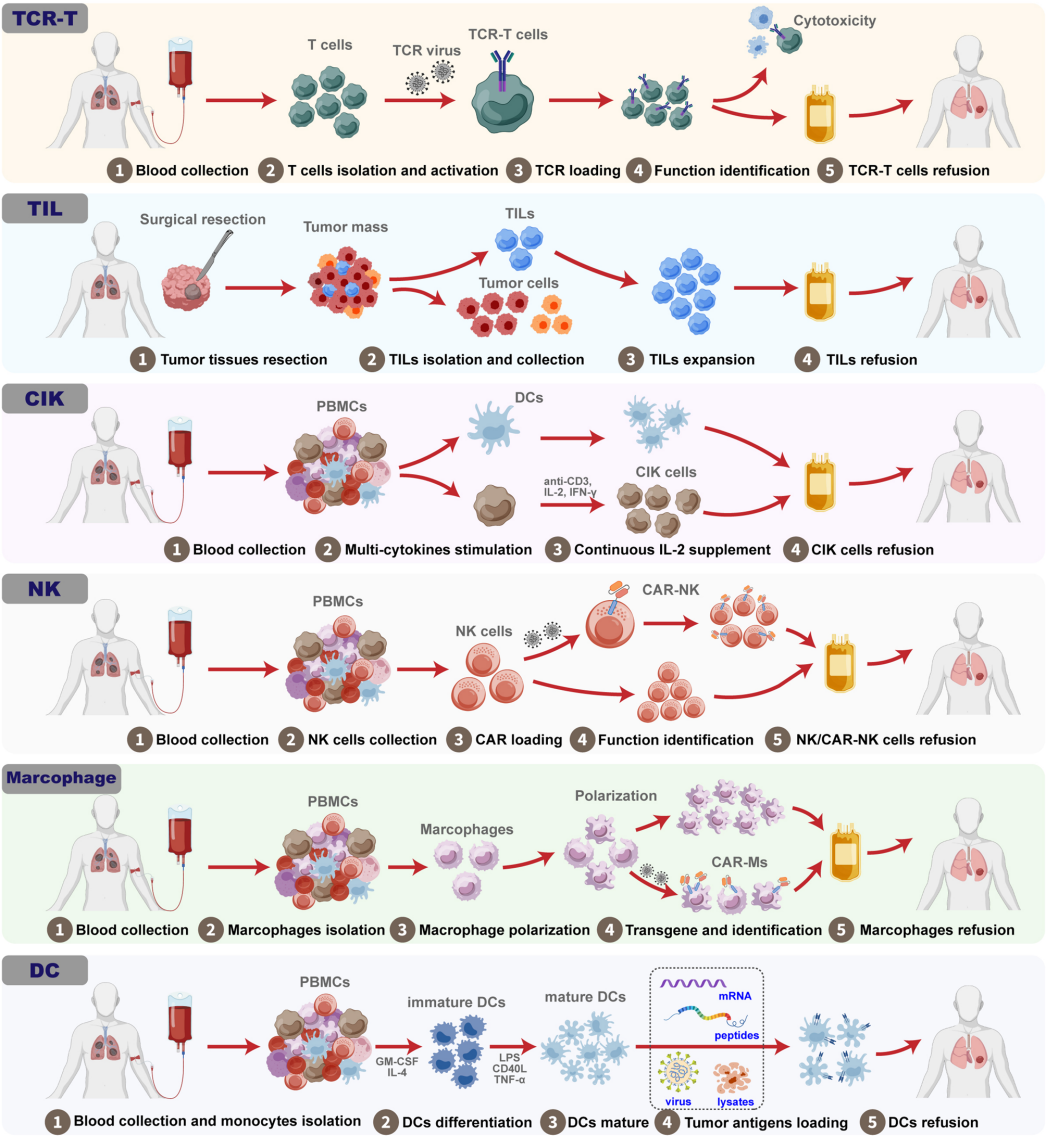
The schema of other cell-based immunotherapies for lung cancer, including TCR-T cells, TILs, CIK cells, NK cells, macrophages, and DCs

As the above summary, CAR-T therapy has attached much attention and occupied the main adoptive cellular immunotherapy applied to treat lung cancer. Notably, other cell-based immunotherapies have also been explored, increasing the options for lung cancer treatment. In this section, we provide an overview of recent advances in other cellular immunotherapies, including TCR-T cells, tumor-infiltrating lymphocytes (TILs), cytokine-induced killer (CIK) cells, NK cells, macrophages, and dendritic cells (DCs) (Fig. 3), listed in Table 3.

**Table 3:** Immune cell-based immunotherapies for lung cancer

Immune cell-based immunotherapy	Targets	Study type	Patient type	Characteristics
**TCR-T cells**	**NY-ESO-1**	clinical trial (NCT02588612)	Patients with NY-ESO-1 positive advanced metastatic NSCLC.	The TCR-T cells were used as second line treatment after completing lymphodepleting chemotherapy and showed primary efficacy and safety.
		clinical trial (NCT03029273)	Patients with advanced NSCLC.	This study aimed to investigate the safety and tolerability of autologous NY-ESO-1 TCR-T cell therapy in subjects with NSCLC who have received prior therapy for their disease but their disease has progressed or relapsed.
		clinical trial (NCT03709706)	Patients with NY-ESO-1/​LAGE-1a-positive advanced NSCLC either alone or in combination with pembrolizumab.	This trial aimed to evaluate safety and tolerability of NY-ESO-1 TCR-T cell therapy with or without pembrolizumab in participants with NSCLC. The study was recently terminated due to reasons pertaining to feasibility.
	**KK-LC-1**	clinical trial (NCT05483491)	Patients with metastatic cancers that express KK-LC-1, including gastric, breast, cervical, and lung cancer.	The safety profile and clinical response of LL-LC-1 TCR-T cell therapy would be determined.
		clinical trial (NCT05035407)	Patients with gastric, breast, cervical, lung, and other KK-LC-1 positive epithelial cancers.	The purpose of this study is to determine the safety of different doses of KK-LC-1 TCR-T cells plus aldesleukin to treat metastatic or refractory/recurrent KK-LC-1 positive cancers.
		clinical trial (NCT03778814)	Patients with KK-LC-1 expression in advanced lung cancer or other solid tumors and matched MHC-A11 typing.	The safety, tolerance and preliminary efficacy of the TCR-T cell immunotherapy on human would firstly be assessed.
**TILs**	**NSCLC**	clinical trial (NCT03215810)	Patients with advanced NSCLC.	The trial was planned to study the safety, side effects, and benefits of TILs when they are given with the drug nivolumab. 20 patients with advanced NSCLC following initial progression on nivolumab monotherapy and the study showed positive safety and clinical activity.
		clinical trial (NCT04614103)	Patients with metastatic NSCLC.	23 patients with NSCLC have accepted autologous TIL therapy and the ORR and disease control rate of patients with NSCLC after treatment reached 26.1% and 82.6%, respectively.
		clinical trial (NCT05566223)	Patients with metastatic NSCLC.	The clinical trial aimed to assess the safety and efficacy of genetically-engineered TIL in which the intracellular immune checkpoint CISH has been inhibited using CRISPR gene editing for the treatment of metastatic NSCLC.
	**Metastatic ** **cancer**	preclinical study pharmacologic inhibition of AKT	An immunodeficient mouse model for cell-based immunotherapy	Pharmacologic inhibition of Akt showed a novel immunometabolomic approach to enhance the persistence of TILs and improve the efficacy of cell-based immunotherapy for metastatic cancer.
**CIK cells**	**SCLC**	case report	An adult male patient with SCLC.	The patient survived for 13 years through a combination of chemotherapy, radiotherapy, and CIK immune cell therapy. It suggested that that CIK cell immunotherapy combined with chemotherapy can prolong patient survival in cases of extensive SCLC.
	**NSCLC**	clinical trial (NCT03987867)	Patients with stage IIIB/IIIC/IV NSCLC.	Autologous CIK cells in combination with a monoclonal antibody against PD-1 (sintilimab) plus chemotherapy was well tolerable and showed encouraging efficacy in patients with advanced NSCLC.
	**NSCLC** **+SCLC**	Meta-Analysis of 16 randomized controlled trials	1197 patients with lung cancer such as NSCLC and SCLC from China.	CIK therapy could increase the ORR, the disease control rate, and the 1-year and 2-year overall survival rate.
		retrospective study	68 patients with lung cancer.	The overall survival rates of patients received combination of chemotherapy and CIK treatment were significantly improved compared to the overall survival rates of patients only received chemotherapy.
**NK cells**	**NSCLC**	clinical trial (NCT02843815)	Patients with advanced NSCLC.	Allogenic NK cells combined with cryosurgical treatment for advanced NSCLC have a synergistic effect, not only enhancing the immune function of patients, improving the quality of life, but significantly increasing the response rate and disease control rate compared to cryoablation group.
		clinical trial (NCT03958097)	Patients with stage IIIB/IIIC or IV NSCLC.	The autologous NK cells did not add extra adverse events and autologous NK therapy plus sintilimab showed promising antitumor activity and an acceptable safety profile in advanced NSCLC patients who failed on the first line treatment.
	**SCLC**	clinical trial (NCT05507593) **DLL3 CAR-NK cells**	Patients with relapsed and refractory extensive SCLC.	It is a multicenter, open-label, phase I clinical trial aimed to evaluate the safety and efficacy of DLL3 CAR-NK cells treatment for relapsed and refractory extensive SCLC.
		preclinical study **DLL3 CAR-NK cells**	In vitro and in vivo mouse tumor models of SCLC.	DLL3 CAR-NK exhibited significant cytotoxicity and cytokine production in vitro and in subcutaneous tumor models of SCLC.
	**NSCLC**	preclinical study **c-Met CAR-NK cells**	In vitro and in vivo mouse tumor models of NSCLC.	c-Met CAR-NK showed a promising strategy for the treatment of c-Met positive NSCLC.
		clinical trial (NCT06454890) **Trop2 CAR-NK cells**	Patients with relapsed/​refractory NSCLC.	It is a single-center, open-labeled, single-arm, non-randomized investigator-initiated trial evaluating the efficacy and safety of Trop2 U-CAR-NK cells therapy combined with chemotherapy for relapsed/refractory NSCLC.
		clinical trial (NCT02839954) **MUC1 CAR-pNK cells**	Patients with MUC1 positive relapsed or refractory solid tumor.	The purpose of this study is to evaluate the safety and effectiveness of MUC1 CAR-pNK cell immunotherapy in patients with MUC1 positive relapsed or refractory solid tumor, including NSCLC.
		preclinical study **NKG2D CAR-NK cells**	In vitro and in vivo mouse tumor models of NSCLC.	IL-21 effectively increased the cytotoxicity of NKG2D CAR-NK cells against lung cancer cells in a dose-dependent manner and suppressed tumor growth in vitro and in vivo.
**Macrophages**	**Lung cancer**	preclinical study TAMs regulated by a CSF1R inhibitor	An orthotopic immunocompetent mouse model of primary lung cancer.	A small molecule CSF1R inhibitor induced a significant reduction of M2 TAMs and an increase of M1 TAMs. When it combined with cisplatin, a significant decrease in tumor burden was observed.
	**NSCLC**	preclinical study Macrophages regulated by CD47 block	A metastatic NSCLC xenograft mouse model	Targeting CD47 could trigger macrophage-mediated elimination of the relapsed NSCLC cells, eliciting synergistic antitumor effect.
	**HER2**	clinical trial (NCT04660929) **HER2 CAR-Ms**	Patients with HER2 overexpressing solid tumors	The trial aimed to investigate the safety and efficacy of HER2 CAR-Ms therapy for the treatment of HER2 overexpressing solid tumors, including NSCLC and SCLC.
**DCs**	**Lung cancer**	clinical trial (NCT02956551) **Personalized DC vaccine**	Patients with lung cancer.	A total of 12 patients were enrolled in this study. Personalized neoantigen peptide-pulsed autologous DC vaccines showed 25% of objective effectiveness, 75% of disease control rate, and nearly 8 months of median overall survival.
		case report	A patient with unresectable advanced lung cancer.	The tumor size decreased significantly after combined therapy with the dendritic cells pulsed with Wt1 and Mucin1 vaccine and erlotinib. The patient has living for more than 600 days without recurrence or metastasis.

### TCR-T cells

Similar to CAR-T therapy, TCR immunotherapy employs T cells to target and recognize tumor antigens through genetic modification with tumor antigen specific T cell receptors [190]. The recognition of specific tumor by TCR-T cells requires the presentation of an HLA or an antigen-presenting cell (APC). Unlike CAR-T cells, TCR-T cells are capable of recognizing intracellular antigens, providing more options for targets and making TCR-T therapy more suitable for the treatment of solid tumors [191]. Multiple researches and clinical trials investigating the anti-tumor efficiency and safety of TCR-T cells for the treatment of lung cancer are underway.

In a clinical trial (NCT02588612), autologous NY-ESO-1 TCR-T cells, called letetresgene autoleucel (lete-cel; GSK3377794) were used for patients with NY-ESO-1 positive advanced metastatic NSCLC as second line treatment after completing lymphodepleting chemotherapy. Lete-cel has showed primary efficacy and safety in patients with advanced and metastatic myxoid/round cell liposarcoma, which implies potential efficacy for the treatment of lung cancer [192]. Two other clinical trials have assessed the efficacy of NY-ESO-1 TCR-T cells in patients with progressed or relapsed NSCLC (NCT03029273 and NCT03709706). In addition to NY-ESO-1, KK-LC-1 is another ideal antigen for TCR-T cells for the treatment of lung cancer [193]. Three clinical studies are underway to determine the safety of applying KK-LC-1 TCR-T cells with aldesleukin to enhance the durability of TCR-T cells after refusion (NCT05483491, NCT05035407, and NCT03778814). Actually, TCR-T cells have achieved breakthroughs and outstanding results in several clinical trials, including trials for pancreatic cancer, synovial sarcoma, and advanced soft tissue sarcoma [194–196]. To apply TCR-T cell therapy for the treatment of lung cancer, preclinical testing to identify a suitable antigen-specific TCR sequence is needed.

### TILs

TIL therapy was first proposed in 1986. Rosenberg SA et al. reported that lymphocytes existed in the tumor tissues of patients with advanced melanoma and demonstrated that these tumor-infiltrating lymphocytes could specifically recognize tumor cells and that refusion into patients enable to eliminate the tumor tissue [197]. The production of TILs for therapeutic applications includes four steps: (1) Tumor tissues resection; (2) TILs isolation and collection; (3) TILs expansion and function identification; (4) TILs refusion into patients. To obtain a sufficient number of TILs, IL-2 is used to stimulate the proliferation of TILs in vitro. The first clinical test showed 55% complete remission in 20 patients with melanoma and revealed the great potential of TIL therapy for oncotherapy [198]. Subsequently, TIL therapy has been used in cervical cancer, colorectal cancer, and ovarian cancer [199]. More than 70 clinical trials of TIL therapy for the treatment of lung cancer have been registered and implemented. In a single-arm open-label phase I trial (NCT03215810), autologous TILs were used for 20 patients with advanced NSCLC following initial progression on nivolumab monotherapy and the study showed positive safety and clinical activity [200]. In recently released data from another clinical (NCT04614103), the ORR and disease control rate of patients with NSCLC after treatment reached 26.1% and 82.6%, respectively. However, several challenges limit the efficacy of TIL therapy, including complex preparation techniques for isolating TILs and the poor durability and persistence of TILs after refusion [201]. Several treatment strategies have been designed and adopted to optimize TIL therapy. Before refusion, pre-lymphodepletion in patients can eradicate immunosuppression and enhance the TILs proliferation and activity [202]. Pharmacologic inhibition of AKT has been demonstrated to enable the expansion of TILs with memory T cell characteristics and prolong the persistence of TILs after adoptive transfer, thereby resulting superior antitumor immunity [203]. In a recent clinical trial (NCT05566223) using CISH as the intracellular checkpoint target, researchers propose to knock out CISH by CRISPR system in TILs from patients with metastatic NSCLC in order to evaluate the safety and efficacy of genetically engineered TIL therapy. Overall, TIL therapy has represented enormous potential for the treatment of lung cancer and other solid tumors, and further fundamental studies and clinical trials are anticipated to optimize TIL therapy.

### CIK cells

Similar to TIL, CIK therapy was proposed more than 30 years. The manufacturing process of CIK cells is relatively simple, including four steps: (1) PBMC isolation from whole blood; (2) Multi-cytokines stimulation (anti-CD3 antibody, IL-2, IFN-γ, etc.); (3) Continuous IL-2 supplementation for CIK cells proliferation; (4) CIK cells refusion into patients [204, 205]. CIK cells are a mixture of CD3 + T cells, NK cells, and NK-like T cells (CD3 + CD56+) (NKT cells). NKT cell subset is the core of CIK and primarily exerts anti-tumor activity by three pathways: (1) CIK cells possess polyclonal TCRs that allow them to recognize tumor cells via TCR-MHC interactions; (2) CIK cells express NK cell activating receptors (NKG2D, DNAM-1, and Nkp30) that allow them to recognize tumor cells without MHC limitation; (3) Some CIK cells express FCγRIII CD16, which allows them to recognize specific tumor cell antigens [206]. After recognizing tumor cells, CIK cells enable to trigger cytotoxicity via releasing granzyme and perforin, secreting killing cytokines (IFN-γ and TNF-α), inducing tumor cells apoptosis via Fas-FasL pathway, and CD16-induced perform antibody-dependent cell-mediated cytotoxicity (ADCC) [204]. CIK therapy has been applied in clinical trials, in which over 5000 patients with more than 30 different types of cancer have been treated [207]. These clinical data revealed that CIK therapy significantly improved the median progression-free and overall survival time. Notably, CIK therapy has achieved remarkable clinical efficacy, and a recent case report showed that a patient with extensive SCLC has survived more than 13 years through a combination of CIK therapy and chemotherapy, demonstrating the high-efficiency and long-term anti-tumor performance of CIK therapy [208]. In addition, the application of CIK cells has also been combined with DCs, which could load tumor-associated antigens, enhance the anti-tumor activity of CIK cells, and induce immunologic memory [209]. In a meta-analysis of 16 randomized controlled trials on the clinical efficacy and safety of about CIK therapy for the treatment of lung cancer, the application of CIK cells improved the ORR, the disease control rate, and the 1-year and 2-year overall survival rate [210]. Moreover, the synergistic effects of immune checkpoint inhibitors and CIK cells have been shown to increase the anti-tumor potency in several preclinical and clinical studies [211–213]. These results suggest that combining CIK cells with other cancer therapies could improve the clinical performance. Although many clinical researches have confirmed its efficiency, CIK cell therapy is still an understudied adoptive cellular immunotherapy, and many challenges need to be overcome, such as the need for a large number of CIK cells for a single refusion, the unexplained association between CIK cells and the TME, and the unknown key factors underlying the clinical efficacy of CIK cell therapy.

### NK cells

As the body’s first responders and resisters, NK cells perform crucial functions in the innate immunosurveillance of viruses, bacteria and cancers [214]. They can rapidly recognize and eradicate abnormal cells through direct cytotoxic reactions and activate other immune cells to initiate adaptive responses. NK cells can be derived and augmented from distinct sources, such as PBMCs, cord blood, induced pluripotent stem cells (iPSCs), hematopoietic stem and progenitor cells (HSPCs), and even the immortalized cell line NK92, which could serve as a guaranteed source for NK cell-based tumor immunotherapy [215, 216]. NK cells have been used for the clinical treatment of multiple tumors. In a phase I trial (NCT02030561), 22 patients with refractory HER2 + solid tumors received trastuzumab and autologous NK cells that were derived from PBMCs and expanded over 10 day coculture with feeder cells, and the result showed that the combination of NK cells and trastuzumab was well tolerated and had preliminary antitumor activity [217]. According to recent updated clinical data, NK cell therapy has demonstrated significant potential and advancements for the treatment of solid tumors and hematologic malignancies when applied in combination with several cytokines and immune checkpoint inhibitors. NK therapy has been used for the treatment of lung cancer, and the combination of an anti-PD-1 antibody with autologous NK cells for the treatment of patients with stage IIIB/IIIC or IV NSCLC has revealed promising antitumor activity and an acceptable safety profile (NCT03958097) [218]. In addition, the safety and efficacy of allogenic NK cells have been preliminarily verified when combined with cryosurgery for the treatment of advanced NSCLC (NCT02843815) [219]. To improve the anti-tumor efficacy, NK cells have been armed with a CAR structure similar to that used for CAR-T therapy. Compared with CAR-T cells, CAR-NK cells have several unique advantages: (1) CAR-NK enables the recognition of tumors via diverse ligand-receptor interaction, not only CAR signals; (2) Without the risk of GvHD, allogenic CAR-NK cells can be directly used for clinical treatment; (3) Limited secretion of inflammatory cytokines reduces the occurrence of CRS in CAR-NK cells [220]. These characteristics illustrate the huge potential of CAR-NK therapy. And it has been verified that the CAR structure and tumor antigen targets are universal for T and NK cells. Recent researches have demonstrated the sufficient anti-tumor efficacy of CAR-NK therapy in hematologic malignancies and solid tumors [36, 221]. DLL3 specific CAR-NK92 exhibited significant cytotoxicity and cytokine production in vitro and in subcutaneous tumor models of SCLC [222]. Another study reported that c-Met CAR-NK cells constructed with the DAP10 domain performed favorable NK cell activation and exerted prominent tumor-inhibitory effects in a xenograft lung adenocarcinoma model [223]. Co-expressing IL-21 and NKG2D enhanced the efficacy of CAR-NK cells for the treatment of lung cancer in vitro and in vivo by activating the AKT signaling pathway [224]. Although these studies demonstrated the efficacy of CAR-NK therapy for lung cancer, the clinical trials only just begun (NCT06454890, NCT05507593, and NCT02839954). Similar to CAR-T cells, CAR-NK cells also face several challenges, such as immunosuppressive TME, poor persistence, and antigen target escape [215, 220]. Future advancements and researches are urgently needed to overcome these obstacles and enhance the therapeutic potential of CAR-NK cell therapy for lung cancer patients.

### Macrophages

Macrophages are an integral component of the innate immune system and can maintain tissue homeostasis via the phagocytosis of pathogens and pathological products [225]. Macrophages exhibit high plasticity and polarize into two diverse functional phenotypes: activated M1 macrophages and alternatively activated M2 macrophages, induced by different stimulating factors [226, 227]. M1 macrophages are normally induced via Toll-like receptors (TLRs) and are mainly responsible for eliminating pathogens and secreting cytokines to activate other immune cells for inflammatory responses. M2 macrophages are induced by IL-4, IL-10, and IL-13, and act to reduce the secretion of pro-inflammatory factors and inhibit inflammatory responses [228]. The two diverse macrophages could transform into each other and cooperate to maintain tissue homeostasis. Moreover, tumor associate macrophages (TAMs) are recruited into the TME, and are involved in the tumor development. Early studies suggested that TAMs perform a role in tumor suppression. However, further researches have shown that tumors enable induce TAMs to the M2 subset to inhibit immunoreaction, induce angiogenesis, and ultimately promote tumor progression. Increased abundance of the M2 subset has been demonstrated to be associated with an unfavorable prognosis of tumor patients. Therefore, TAMs have been regarded as a target for tumor diagnosis and treatment, with the main strategies being inhibition of TAM recruitment and proliferation and regulation of TAM polarization from the M2 subset to the M1 subset [229]. The colony stimulating factor 1 - colony stimulating factor1 receptor (CSF1-CSF1R) axis is a pivotal pathway for M2 TAM recruitment and durability. Blocking the CSF1-CSF1R axis has been verified to induce TAM elimination and depolarization [230]. In a preclinical lung cancer model, a CSF1R inhibitor was shown to regulate TAMs and lead to a significant decrease in tumor burden when combined with first-line chemotherapy [231]. The innate immunosuppressive regulator signal regulatory protein alpha (SIRPα) and its ligand CD47 are upregulated in NSCLC tumors and this axis inhibits the anti-angiogenic therapy. Based on it, recent research has shown that blocking CD47 could increase macrophage infiltration and sensitize NSCLC tumors to anti-angiogenic therapy, providing a novel therapic strategy for the treatment of NSCLC through disrupting of the CD47/SIRPα interaction and angiogenetic axis [232]. In addition to directly targeting TAMs, macrophage antitumor cytotoxicity can also develop from remolding macrophages to have a CAR structure. Initial studies demonstrated that CAR-macrophages (CAR-Ms) performed antigen-specific phagocytosis and tumor elimination in vitro and in vivo tumor mouse models [233–236]. Subsequent researches added two intercellular domains to CAR-Ms, individually responsible for phagocytosis and proinflammatory reactions, significantly enhancing the anti-tumor efficacy [237]. Compared with CAR-T and CAR-NK cells, CAR-Ms possess the best homing and infiltration abilities, which are crucial for solid tumors treatment. Moreover, CAR-Ms can also enhance the antigen presentation to further induce an adaptive immune response [238]. Overall, as two macrophage-targeted therapies, both TAMs and CAR-Ms showed vital promising for solid tumors treatment. A phase I clinical trial about CAR-Ms for the treatment of HER2 + solid tumors is ongoing (NCT04660929) [239].

### DCs

DCs are a type of multifunctional and efficient APCs, which could recruit and activate T cells for targeted tumor clearance ability in adaptive immunity [240]. When facing tumor cells, DCs could recognize and present the tumor antigen to T cells via the peptide-major histocompatibility complex (pMHC) as the first activation signal. Meanwhile, the costimulatory molecules (CD40, CD80, and CD86) in DCs surface would further promote T cell proliferation and mature as the second activation signal [240, 241]. Moreover, DCs could secrete various cytokines and chemokines to assist and regulate immune responses and anti-tumor effects. DCs have been designed and used for tumor immunotherapy based on its antigen-presenting ability, called as DC vaccines [242]. The production process of DC vaccines includes five steps: (1) Monocytes isolate and acquirement; (2) Multi-cytokines (GM-CSF and IL-4) stimulation for DCs differentiation; (3) Inducible factors (LPS, CD40L, and TNF-α) stimulation for DCs mature; (4) Tumor antigens loading by tumor cell lysates, recombinant peptides, virus, or mRNA; (5) DCs refusion into patients [242]. DC vaccines have been used in clinical researches for more than 30 years. Initial clinical trials revealed the security and immunogenicity for tumor patients. The first DC vaccine product (provenge) was approved by the FDA for advanced prostate cancer in 2010, in which the DCs were activated by prostatic acid phosphatase, a typical prostate cancer antigen [243]. There have been more than 100 clinical trials about DCs for lung cancer. A recent report treating lung cancer patients with personalized neoantigen peptide-pulsed autologous DC vaccines showed 25% of objective effectiveness, 75% of disease control rate, and nearly 8 months of median overall survival. It presented a new therapeutic opportunity for the treatment of lung cancer by neoantigen vaccine therapy (NCT02956551) [244]. Moreover, DC vaccines were also combined with targeted drugs, immune checkpoint inhibitors, and other cell-based therapies. A recent study reported that DC vaccination in combination with erlotinib eliminated the tumor in a patient with inoperable lung adenocarcinoma, without recurrence or metastasis more than 600 days [245]. These exciting results suggested that DC vaccines have great potential in lung cancer treatment and inhibiting recurrence.

## Combination immunotherapy strategies in lung cancer

As discussed above, a series of cell-based immunotherapies have revolutionized the treatment of lung cancer and various solid tumors and presented huge potential for clinical application. However, the heterogeneity of lung cancer and the complexity of the TME are important factors limiting the monotherapy efficacy and cannot be neglected [246, 247]. Therefore, combination immunotherapy strategies have been gradually explored and adopted for lung cancer in order to improve patient survival time.

### Immune checkpoint inhibitors

The application of immune checkpoint inhibitors that block the CTLA-4, PD-1, and PD-L1 has achieved remarkable clinical efficacy for NSCLC patients. Moreover, these inhibitors have been combined with cellular immunotherapies [25]. A clinical report showed that the treatment efficacy of autologous CIK cells after treatment with PD-1 blocking antibodies (pembrolizumab and nivolumab) in patients with advanced NSCLC was enhanced [248]. When applied in combination with CAR-T cells, an anti-PD-1 antibody potently improved CAR-T therapy efficacy by eradicating tumors without severe toxicity [97, 138, 249].

### Anti‑angiogenic agents

Blocking vascular endothelial growth factor (VEGF) signaling could normalize tumor vessels in various tumors and anti‑angiogenic agents have been approved for the treatment of NSCLC and significantly decrease the mortality risk [250]. An early study revealed that anti‑angiogenic agents increase lymphocyte infiltration into tumor and enhance the effectiveness of adoptive immunotherapy in mouse tumor model. Moreover, anti‑angiogenic agents have been combined with CAR-T therapy and the preclinical research has shown that anti-mouse VEGF antibodies enhance CAR-T cell infiltration, inhibit tumor growth, and prolong the survival time of GBM-bearing mice compared with EGFRvIII-CAR-T cell therapy alone [251].

### Cancer vaccines

Vaccines have been gradually applied to treat cancer, promoting antigen-specific immune responses by introducing tumor antigens to stimulate the host immune system. A recent phase II clinical trial (NCT05077709) shows that applying a cancer vaccine (IO102-IO103) targeting IDO and PD-L1 along with an anti-PD-1 antibody (keytruda) has promising clinical effects on patients with metastatic NSCLC, with a disease control rate of more than 80% [252]. Vaccines have also been combined with CAR-T therapy. An RNA vaccine, consisting of CLDN6 ribonucleic acid lipoplexes, administered alongside CLDN6 CAR-T cells in 21 patients with CLDN6 + relapsed or refractory advanced-stage solid tumors resulted in a disease control rate of more than 60% (NCT04503278) [253]. This preliminary success highlights the potential of using cancer vaccines to enhance the persistence of CAR-T cells.

### Gut microbiota

Recent evidence shows that gut microbiota influence the host immune system, including immune cell development, differentiation, and function [254]. Metabolites are the main way in which the gut microbiota interacts with the host. Tanoue et al. isolated bacterial strains from healthy human donor faeces and colonized them into mice, which enhanced the therapeutic efficacy of immune checkpoint inhibitors in syngeneic tumor models [255]. Bachem et al. explored the interactions between the microbiota and ACT and revealed that microbiota-derived SCFAs could promote CAR-T cell durability and anti-tumor effect [256]. This study suggested that microbial metabolites guide the metabolic rewiring of activated CAR-T cells. Overall, combination therapy represents a new strategy for improving cellular immunotherapies and better understanding differences in clinical efficacy among patients.

## Conclusion

Adoptive cellular immunotherapy has rapidly developed and fundamentally altered the options for cancer treatment. This breakthrough approach has been used in clinical settings to treat various types of cancer [27]. For lung cancer, traditional treatment strategies, including surgical resection, chemotherapy, radiotherapy and targeted therapy, cannot achieve brilliant clinical effects for every patient [7]. However, multiple cellular immunotherapies have demonstrated promising results not only in mouse models but also in clinical trials, which is encouraging for patients with refractory and relapsed lung cancer. However, cellular immunotherapies still face several barriers and challenges that limit their efficacy and further clinical application [38]. In this review, we systematically summarize and analyze the evolution and application of cellular immunotherapies, including CAR-T cells, TCR-T cells, TILs, CIK cells, NK cells, macrophages, and DCs, as well as several optimized measures and combination immunotherapy strategies, for the treatment of lung cancer.

CAR-T therapy has achieved outstanding clinical responses in the treatment of hematological malignancies and displayed great promise for solid tumors [36]. A series of antigens targeting surface proteins, including EGFR, CEA, MSLN, HER2, ROR1, MUC1, and DLL3 in lung carcinoma cells have been incorporated into CAR structure to allow CAR-T cells target lung cancer tissue. CAR-T cells targeting EGFR, CEA, and MSLN have been carried out for patients with lung cancer in clinical trials. Preliminary results show the sufficient safety and efficacy of CAR-T cells against lung cancer. The heterogeneity and complexity of lung cancers further impel the exploration of superior targets. In addition, the sterile TME is another dominant factor that inhibits the cytotoxicity and clinical performance of CAR-T cells in solid tumors. Various approaches and strategies have been adopted in vitro and in vivo to improve the efficacy of CAR-T therapy, with a focus on improving the security, enhancing the cytotoxicity, and increasing the durability [157]. Several recent studies have demonstrated that the repurposing of traditonal drugs for CAR-T therapy is attractive and some have revealed potential for clinical application. Moreover, other cell-based immunotherapies, including TCR-T cells, TILs, CIK cells, NK cells, macrophages, and DCs, have also enriched the treatment choices for patients with lung cancer.

Taken together, cellular immunotherapies for lung cancer have greatly developed and yielded promising preliminary results. With the continuous optimization of treatment strategies and protocols, cellular immunotherapies are expected to become a major treatment for patients with lung cancer.

## Data Availability

No datasets were generated or analysed during the current study.

## Abbreviations

WHO: World Health Organization
SCLC: small cell carcinoma
NSCLC: non-small cell carcinoma
PD-1: programmed death-1
PD-L1: programmed death-ligand 1
CAR-T: chimeric antigen receptor T
CR: complete remission
TME: tumor microenvironment
TCR: T cell receptor
TAA: tumor-associated antigen
EGFR: epidermal growth factor receptor
CEA: carcinoembryonic antigen
MSLN: mesothelin
HER2: human epidermal growth factor receptor 2
ROR1: receptor tyrosine kinase-like orphan receptor 1
MUC1: Mucin 1
PSCA: prostate stem cell antigen
HER: human epidermal growth factor receptor
DLL3: delta-like ligand 3
CXCR5: C-X-C Chemokine receptor type 5
SMAD7: mothers against decapentaplegic homologue 7
HMGB1: high mobility group protein B1
CLL: chronic lymphocytic leukemia
mAb: monoclonal antibody
TNBC: triple negative breast cancer
ADC: antibody-drug conjugate
PTK7: protein tyrosine kinase 7
OTOTT: on-target off-tumor toxicity
CRS: cytokine release syndrome
CEACAM5: carcinoembryonic antigen related cell adhesion molecule 5
PTP1B: protein tyrosine phosphatase 1B
CISH: SH2-containing protein
NK: natural killer
A2AR: adenosine A2A receptor
TGFBR: TGF-β receptor
T_N_ cell: naïve T cell
T_SCM_ cell: stem cell memory T cell
T_CM_ cell: central memory T cell
T_EM_ cell: effector memory T cell
T_EFF_ cell: effector T cell
PBMC: peripheral blood mononuclear cells
NHL: non-Hodgkin lymphoma
SCFA: short-chain fatty acids
DON: 6-diazo-5-oxo-L-norleucine
NAC: N-acetylcysteine
TET2: tet methylcytidine dioxygenase 2
HDACi: class I histone deacetylase inhibitors
UA: urolithin A
TIL: tumor-infiltrating lymphocytes
CIK: cytokine-induced killer
APC: antigen-presenting cell
ORR: objective response rate
ADCC: antibody-dependent cell-mediated cytotoxicity
iPSC: induced pluripotent stem cell
HSPC: hematopoietic stem and progenitor cell
TLR: Toll-like receptor
TAM: tumor associate macrophage
CSF1-CSF1R: colony stimulating factor 1 - colony stimulating factor1 receptor
SIRPα: signal regulatory protein alpha
CAR-M: CAR-macrophage
VEGF: vascular endothelial growth factor
